# Supplementary material for: Triplon band splitting and topologically protected edge states in the dimerized antiferromagnet
Source: Nat Commun. 2019 May 8;10:2096. doi: 10.1038/s41467-019-10091-6 (PMC6506493; doi:10.1038/s41467-019-10091-6)
Supplement: Supplementary file 1 — Supplementary Information [file 41467_2019_10091_MOESM1_ESM.pdf]

Triplon band splitting and topologically protected edge states in the dimerized antiferromagnet

Kazuhiro Nawa[1],* Kimihiko Tanaka[2], Nobuyuki Kurita[2], Taku J Sato[1], Haruki Sugiyama[3], Hidehiro Uekusa[3], Seiko Ohira-Kawamura[4], Kenji Nakajima[4], and Hidekazu Tanaka[2]†
[1]*Institute of Multidisciplinary Research for Advanced Materials, Tohoku University, 2-1-1 Katahira, Sendai 980-8577, Japan*
[2]*Department of Physics, Tokyo Institute of Technology, Meguro-ku, Tokyo 152-8551, Japan*
[3]*Department of Chemistry, Tokyo Institute of Technology, Meguro-ku, Tokyo 152-8551, Japan*
[4]*Materials and Life Science Division, J-PARC Center, Tokai, Ibaraki 319-1195, Japan*

SUPPLEMENTARY NOTE 1. CRYSTAL STRUCTURE.

Supplementary Figure 1 shows the redetermined crystal structure of $Ba_2CuSi_2O_6Cl_2$. The structure is closely related to that of $Ba_2CoSi_2O_6Cl_2$ [1]. The crystal structure has a CuO_4Cl pyramid feature with a Cl^- ion on an apex. The CuO_4Cl pyramids are linked via SiO_4 tetrahedra in the *ab* plane. Magnetic spin-1/2 Cu^{2+} is located at the center of the base composed of O^{2-}, which is parallel to the *ab* plane. Two neighboring CuO_4Cl pyramids along the *c* axis are placed with their bases facing each other. The CuO_4Cl pyramids are linked via SiO_4 tetrahedra in the *ab* plane. The atomic linkage in the *ab* plane is approximately the same as that of $BaCuSi_2O_6$ [2, 3].

It is natural to assume from the crystal structure that two Cu^{2+} spins located on the bases of neighboring CuO_4Cl pyramids along the *c* axis form an antiferromagnetic dimer, and the dimers are coupled by weak exchange interactions in the *ab* plane. In fact, the presented excitation spectrum supports this model. The exchange network of $Ba_2CuSi_2O_6Cl_2$ is illustrated in Supplementary Fig. 1c. In the original crystal structure reported in Ref. [4], there is no alternation of the interdimer interactions along the *a* and *b* axes, while in the redetermined structure the interdimer interactions are alternate along the *a* axis. It is also close to a 2D exchange network in $BaCuSi_2O_6$ [3, 5]. However, it should be emphasized that all the dimers are symmetrically equivalent in $Ba_2CuSi_2O_6Cl_2$, while three inequivalent dimers are resolved in $BaCuSi_2O_6$ owing to a structural transition [6].

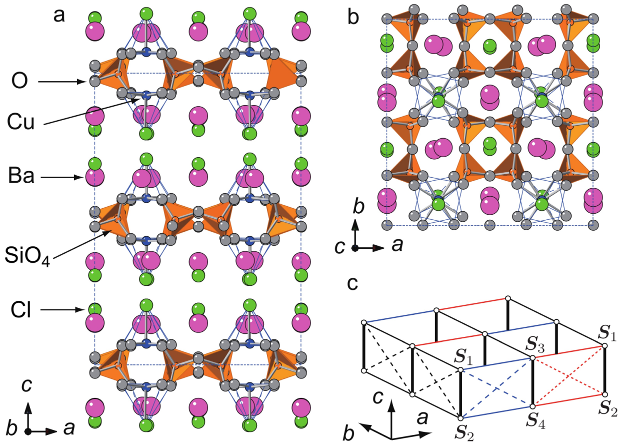

Supplementary Figure 1. **a, b.** Crystal structure of $Ba_2CuSi_2O_6Cl_2$ viewed along (**a**) the *b*-axis and (**b**) the *c*-axes. Dashed lines indicate a unit cell. **c.** 2D model of the exchange network. Thick solid lines represent the intradimer exchange interaction *J*. Thin solid, dashed, and dotted lines represent the interdimer exchange interactions, which are alternating along the *a* axis. Crystallographic data are provided as a Source Data file.

Supplementary Table 1. Crystal data for $Ba_2CuSi_2O_6Cl_2$.

Chemical formula	$Ba_2CuSi_2O_6Cl_2$
Space group	$Cmc2_1$
a (Å)	13.9064(3)
b (Å)	13.8566(3)
c (Å)	19.5767(4)
V (Å^3)	3772.34(14)
Z	16
R; wR ($I > 2\sigma(I)$)	0.0376; 0.0803

Supplementary Table 2. Fractional atomic coordinates ($\times 10^4$) and equivalent isotropic displacement parameters ($\text{Å}^2 \times 10^3$) for $Ba_2CuSi_2O_6Cl_2$.

Atom	x	y	z	U_{eq}
Ba(1)	5000	6058(1)	3535(1)	16(1)
Ba(2)	5000	1445(1)	3547(1)	16(1)
Ba(3)	7283(1)	3787(1)	6398(1)	14(1)
Ba(4)	7715(1)	3684(1)	3587(1)	15(1)
Ba(5)	5000	1005(1)	6436(1)	16(1)
Ba(6)	5000	6383(1)	6459(1)	15(1)
Cu(1)	7496(1)	6219(1)	4252(2)	13(1)
Cu(2)	7494(1)	1250(1)	5736(1)	6(1)
Si(1)	6109(2)	2610(2)	5010(2)	9(1)
Si(2)	6112(2)	4864(2)	4973(2)	8(1)
Si(3)	8889(2)	4854(2)	4978(2)	8(1)
Si(4)	8891(2)	2600(2)	5021(2)	8(1)
O(1)	5000	2433(6)	4761(5)	12(2)
O(2)	6747(5)	2389(5)	4343(4)	13(1)
O(3)	6311(4)	2025(4)	5703(4)	11(1)
O(4)	6241(5)	3746(3)	5219(4)	13(2)
O(5)	6746(5)	5045(5)	4296(4)	13(1)
O(6)	5000	5029(6)	4731(5)	11(2)
O(7)	6323(5)	5495(4)	5650(4)	11(1)
O(8)	8678(5)	5448(5)	4285(4)	16(2)
O(9)	10000	5035(7)	5217(5)	15(2)
O(10)	8264(5)	5073(5)	5649(4)	13(1)
O(11)	8764(5)	3724(4)	4767(4)	15(2)
O(12)	10000	2445(6)	5273(5)	14(2)
O(13)	8692(5)	1975(4)	4346(4)	14(1)
O(14)	8252(5)	2433(5)	5694(4)	14(1)
Cl(1)	7460(4)	6440(4)	2943(3)	50(1)
Cl(2)	5000	6015(4)	1893(3)	35(1)
Cl(3)	5000	1260(3)	1908(5)	65(3)
Cl(4)	5000	1370(5)	8056(4)	51(2)
Cl(5)	7493(3)	1052(4)	7054(3)	48(1)
Cl(6)	5000	6294(3)	8089(4)	59(2)

SUPPLEMENTARY NOTE 2. DECREASE OF THE INTENSITY CENTERED AT 2.6 meV.

To confirm that the decrease of the intensity centered at 2.6 meV is not an extrinsic effect, we checked the data measured at different E_i values. Supplementary Figure 2 shows a color contour map measured at 2.5 K with an E_i of 3.14 meV. The same dispersion relations as those measured with an E_i of 5.9 meV are obtained, indicating that the gap between two triplons bands is intrinsic.

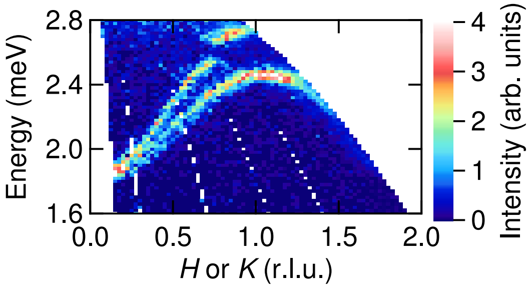

Supplementary Figure 2. Color contour maps of the scattering intensities sliced along the (H, 0) and (0, K) direction. Intensity is integrated along both K with a thickness of $\Delta K = \pm 0.06$ and all collected L values. The presented data are collected at 2.5 K with an E_i of 3.13 meV. Source data are provided as a Source Data file.

SUPPLEMENTARY NOTE 3. DERIVATION OF DISPERSION RELATIONS.

In this section, we start from the model described in Fig. 1a and derive the dispersion relations of triplet excitations. The spin Hamiltonian is given by

$$\begin{aligned}
\mathcal{H} &= \mathcal{H}_0 + \mathcal{H}', \\
\mathcal{H}_0 &= J \sum_m \sum_n \mathbf{S}_{mn1} \cdot \mathbf{S}_{mn2}, \\
\mathcal{H}' &= \sum_{m+n=\text{even}} \sum_{\langle i,j \rangle} \Big(J^{\text{a}}_{ij} \mathbf{S}_{mni} \cdot \mathbf{S}_{(m+1)nj} \\
&\quad + J^{\text{a}'}_{ij} \mathbf{S}_{(m-1)ni} \cdot \mathbf{S}_{mnj} \\
&\quad + J^{\text{b}}_{ij} \mathbf{S}_{mni} \cdot \mathbf{S}_{m(n+1)j} + J^{\text{b}}_{ij} \mathbf{S}_{m(n-1)i} \cdot \mathbf{S}_{mnj} \Big),
\end{aligned} \tag{1}$$

where $\mathcal{H}_0$ represents intradimer exchange terms from J and $\mathcal{H}'$ represents interdimer exchange terms from J^{ξ}_{ij} and $J^{\xi\prime}_{ij}$ ($\xi =$ a, b). $\mathbf{S}_{mni}$ is defined as the i-th Cu atom of the (m, n)-th dimer pair (see Fig. 1b in the main text). Dimers on the two different sublattices are distinguished by m and n: $m+n$ becomes even for one sublattice and odd for the other. For each dimer pair (m, n), a spin operator $\mathbf{S}_{mn}$, $\mathbf{T}_{mn}$ can be defined as

$$\mathbf{S}_{mn} \equiv \mathbf{S}_{mn1} + \mathbf{S}_{mn2}, \quad \mathbf{T}_{mn} \equiv \mathbf{S}_{mn1} - \mathbf{S}_{mn2}. \tag{2}$$

Thus, $\mathcal{H}_0$ is rewritten as

$$\mathcal{H}_0 = \frac{J}{2} \sum_{m+n=\text{even}} \Big(\mathbf{S}^2_{mn} + \mathbf{S}^2_{(m+1)n} - 3 \Big), \tag{3}$$

since $\mathbf{S}^2_{mn1} = \mathbf{S}^2_{mn2} = 3/4$. In addition, $\mathcal{H}'$ can be projected in a subspace constructed by the basis of $\mathcal{H}_0$ as

$$\begin{aligned}
\mathcal{H}' &= \mathcal{H}'_{\text{tt}} + \mathcal{H}'_{\text{ss}}, \\
\mathcal{H}'_{\text{tt}} &= \sum_{m+n=\text{even}} \Big(J^{\text{A}} \mathbf{T}_{mn} \cdot \mathbf{T}_{(m+1)n} \\
&\quad + J^{\text{A}'} \mathbf{T}_{(m-1)n} \cdot \mathbf{T}_{mn} \\
&\quad + J^{\text{B}} \mathbf{T}_{mn} \cdot \mathbf{T}_{m(n+1)} + J^{\text{B}} \mathbf{T}_{m(n-1)} \cdot \mathbf{T}_{mn} \Big), \\
\mathcal{H}'_{\text{ss}} &= \sum_{m+n=\text{even}} \Big(J^{\text{A}}_{\text{s}} \mathbf{S}_{mn} \cdot \mathbf{S}_{(m+1)n} \\
&\quad + J^{\text{A}'}_{\text{s}} \mathbf{S}_{(m-1)n} \cdot \mathbf{S}_{mn} \\
&\quad + J^{\text{B}}_{\text{s}} \mathbf{S}_{mn} \cdot \mathbf{S}_{m(n+1)} + J^{\text{B}}_{\text{s}} \mathbf{S}_{m(n-1)} \cdot \mathbf{S}_{mn} \Big),
\end{aligned} \tag{4}$$

where

$$\begin{aligned}
J^{\text{A}} &= \frac{1}{4}(J^{\text{a}}_{11} - J^{\text{a}}_{12} - J^{\text{a}}_{21} + J^{\text{a}}_{22}), \\
J^{\text{A}'} &= \frac{1}{4}(J^{\text{a}'}_{11} - J^{\text{a}'}_{12} - J^{\text{a}'}_{21} + J^{\text{a}'}_{22}), \\
J^{\text{B}} &= \frac{1}{4}(J^{\text{b}}_{11} - J^{\text{b}}_{12} - J^{\text{b}}_{21} + J^{\text{b}}_{22}), \\
J^{\text{A}}_{\text{s}} &= \frac{1}{4}(J^{\text{a}}_{11} + J^{\text{a}}_{12} + J^{\text{a}}_{21} + J^{\text{a}}_{22}), \\
J^{\text{A}'}_{\text{s}} &= \frac{1}{4}(J^{\text{a}'}_{11} + J^{\text{a}'}_{12} + J^{\text{a}'}_{21} + J^{\text{a}'}_{22}), \\
J^{\text{B}}_{\text{s}} &= \frac{1}{4}(J^{\text{b}}_{11} + J^{\text{b}}_{12} + J^{\text{b}}_{21} + J^{\text{b}}_{22}).
\end{aligned} \tag{5}$$

Dispersion relations are obtained by applying a bond-operator approach [7–9] to Supplementary eqs. (3) and (4). Singlet and triplet creation operators are defined as

$$\begin{aligned} s^\dagger_{mn}|0\rangle &= \frac{1}{\sqrt{2}}(|\uparrow\rangle_{mn1}|\downarrow\rangle_{mn2} - |\downarrow\rangle_{mn1}|\uparrow\rangle_{mn2}), \\ t^\dagger_{xmn}|0\rangle &= -\frac{1}{\sqrt{2}}\,(|\uparrow\rangle_{mn1}|\uparrow\rangle_{mn2} \\ &\qquad - |\downarrow\rangle_{mn1}|\downarrow\rangle_{mn2}), \\ t^\dagger_{ymn}|0\rangle &= \frac{i}{\sqrt{2}}\,(|\uparrow\rangle_{mn1}|\uparrow\rangle_{mn2} + |\downarrow\rangle_{mn1}|\downarrow\rangle_{mn2}), \\ t^\dagger_{zmn}|0\rangle &= \frac{1}{\sqrt{2}}(|\uparrow\rangle_{mn1}|\downarrow\rangle_{mn2} + |\downarrow\rangle_{mn1}|\uparrow\rangle_{mn2}), \end{aligned} \tag{6}$$

so that they follow bosonic commutation relations. In this definition, the number of bosons per dimer is constrained to 1 as

$$s^\dagger_{mn}s_{mn} + \sum_{\alpha=x,y,z} t^\dagger_{\alpha mn}t_{\alpha mn} = 1. \tag{7}$$

Then, the squared operator $\mathbf{S}^2_{mn}$ and α component ($\alpha = x, y, z$) of $\mathbf{S}_{mn}$ and $\mathbf{T}_{mn}$ are given as

$$\begin{aligned} \mathbf{S}^2_{mn} &= \sum_{\alpha=x,y,z} t^\dagger_{\alpha mn}t_{\alpha mn}, \\ S_{\alpha mn} &= -i\sum_{\beta,\gamma=x,y,z} \epsilon_{\alpha\beta\gamma}t^\dagger_{\beta mn}t_{\gamma mn}, \\ T_{\alpha mn} &= s^\dagger_{mn}t_{\alpha mn} + t^\dagger_{\alpha mn}s_{mn}, \end{aligned} \tag{8}$$

where $\epsilon_{\alpha\beta\gamma}$ represents an antisymmetric tensor. At zero field, the ground state is a product of the singlet at each dimer, and thus, the triplon density is zero. Thus, a mean-field approximation that neglects the dynamics of singlet operators should be applicable. By replacing creation and annihilation operators by its expectation value, $\langle s^\dagger_{mn}\rangle \sim \langle s_{mn}\rangle \sim 1$, and neglecting high-order terms, Supplementary eqs. (3) and (4) become

$$\begin{aligned} \mathcal{H}_0 &= J\sum_{m+n=\text{even}}\Big(\sum_{\alpha=x,y,z} t^\dagger_{\alpha mn}t_{\alpha mn} - \frac{3}{4}\Big), \\ \mathcal{H}' &\sim \sum_{m+n=\text{even}}\sum_{\alpha=x,y,z}\Big[J^{\mathrm{A}}\big\{t^\dagger_{\alpha mn}t_{\alpha(m+1)n} \\ &\quad + t^\dagger_{\alpha mn}t^\dagger_{\alpha(m+1)n} + (h.c.)\big\} \\ &\quad + J^{\mathrm{A}'}\big\{t^\dagger_{\alpha(m-1)n}t_{\alpha mn} + t^\dagger_{\alpha(m-1)n}t^\dagger_{\alpha mn} + (h.c.)\big\} \\ &\quad + J^{\mathrm{B}}\big\{t^\dagger_{\alpha mn}t_{\alpha m(n+1)} + t^\dagger_{\alpha mn}t^\dagger_{\alpha m(n+1)} + (h.c.)\big\} \\ &\quad + J^{\mathrm{B}}\big\{t^\dagger_{\alpha m(n-1)}t_{\alpha mn} + t^\dagger_{\alpha m(n-1)}t^\dagger_{\alpha mn} + (h.c.)\big\}\Big]. \end{aligned} \tag{9}$$

A $\mathbf{k}$-dependent form is obtained by Fourier transformation defined at each sublattice as

$$\begin{aligned} t^\dagger_{\alpha mn} &= \sqrt{\frac{2}{N}}\sum_{\mathbf{k}} e^{i\mathbf{k}\cdot\mathbf{r}_{mn}}t^{\dagger,1}_{\alpha\mathbf{k}}, \\ t_{\alpha mn} &= \sqrt{\frac{2}{N}}\sum_{\mathbf{k}} e^{-i\mathbf{k}\cdot\mathbf{r}_{mn}}t^{1}_{\alpha\mathbf{k}}, \end{aligned} \tag{10}$$

for $m+n=$ even and

$$
\begin{aligned}
t^{\dagger}_{\alpha mn} &= \sqrt{\frac{2}{N}}\sum_{\mathbf{k}} e^{i\mathbf{k}\cdot\mathbf{r}_{mn}} t^{\dagger,2}_{\alpha\mathbf{k}},\\
t_{\alpha mn} &= \sqrt{\frac{2}{N}}\sum_{\mathbf{k}} e^{-i\mathbf{k}\cdot\mathbf{r}_{mn}} t^{2}_{\alpha\mathbf{k}},
\end{aligned}
\tag{11}
$$

for $m+n=$ odd, where N describes the number of dimers. This procedure leads to the following quadratic form:

$$
\begin{aligned}
\mathcal{H}_0 &= J\sum_{\mathbf{k}}\Big(\sum_{\alpha=x,y,z} t^{\dagger}_{\alpha\mathbf{k}} t_{\alpha\mathbf{k}} - \frac{3}{4}\Big),\\
\mathcal{H}' &= \sum_{\mathbf{k}}\sum_{\alpha=x,y,z} \Lambda_{\mathbf{k}}\big\{t^{\dagger,1}_{\alpha\mathbf{k}} t^{2}_{\alpha\mathbf{k}} + t^{\dagger,1}_{\alpha\mathbf{k}} t^{\dagger,2}_{\alpha\mathbf{k}} + (h.c.)\big\},
\end{aligned}
\tag{12}
$$

where

$$
\Lambda_{\mathbf{k}} = J^{\mathrm{A}} e^{-ik_x a/2} + J^{\mathrm{A}'} e^{ik_x a/2} + J^{\mathrm{B}}\big(e^{-ik_y b/2} + e^{ik_y b/2}\big). \tag{13}
$$

Supplementary eq. (12) can be described using a 4 × 4 matrix as

$$
\begin{aligned}
\mathcal{H} &= \mathcal{H}_0 + \mathcal{H}' = \frac{1}{2}\sum_{\mathbf{k}}\sum_{\alpha=x,y,z}\mathcal{H}_\alpha - \frac{3}{4}NJ,\\
\mathcal{H}_\alpha &= \Big(t^{\dagger,1}_{\alpha\mathbf{k}} t^{\dagger,2}_{\alpha(-\mathbf{k})} t^{1}_{\alpha\mathbf{k}} t^{2}_{\alpha(-\mathbf{k})}\Big)\,\mathcal{M}_{\mathbf{k}}\begin{pmatrix} t^{1}_{\alpha\mathbf{k}}\\ t^{2}_{\alpha\mathbf{k}}\\ t^{\dagger,1}_{\alpha(-\mathbf{k})}\\ t^{\dagger,2}_{\alpha(-\mathbf{k})}\end{pmatrix},
\end{aligned}
\tag{14}
$$

which is the same as eq. (1) in the main text (except for the omitted constant term), where

$$
\mathcal{M}_{\mathbf{k}} = \begin{pmatrix} J & \Lambda_{\mathbf{k}} & 0 & \Lambda_{\mathbf{k}}\\ \Lambda^*_{\mathbf{k}} & J & \Lambda^*_{\mathbf{k}} & 0\\ 0 & \Lambda_{\mathbf{k}} & J & \Lambda_{\mathbf{k}}\\ \Lambda^*_{\mathbf{k}} & 0 & \Lambda^*_{\mathbf{k}} & J \end{pmatrix}. \tag{15}
$$

The dispersion relation can be obtained by Bogoliubov transformation, which is equivalent to a procedure determining a paraunitary matrix $T_{\mathbf{k}}$ that satisfies

$$
T^{\dagger}_{\mathbf{k}}\mathcal{M}_{\mathbf{k}}T_{\mathbf{k}} = \begin{pmatrix} E_{+,\mathbf{k}} & 0 & 0 & 0\\ 0 & E_{-,\mathbf{k}} & 0 & 0\\ 0 & 0 & E_{+,-\mathbf{k}} & 0\\ 0 & 0 & 0 & E_{-,-\mathbf{k}} \end{pmatrix}. \tag{16}
$$

Owing to orthogonality and completeness of the new basis, $T^{\dagger}_{\mathbf{k}}\Sigma T_{\mathbf{k}} = T_{\mathbf{k}}\Sigma T^{\dagger}_{\mathbf{k}} = \Sigma$, where $\Sigma \equiv \mathrm{diag}(1,1,-1,-1)$. Therefore, Supplementary eq. (16) is equivalent to the relation

$$
\Sigma\mathcal{M}_{\mathbf{k}}T_{\mathbf{k}} = T_{\mathbf{k}}\Sigma\begin{pmatrix} E_{+,\mathbf{k}} & 0 & 0 & 0\\ 0 & E_{-,\mathbf{k}} & 0 & 0\\ 0 & 0 & E_{+,-\mathbf{k}} & 0\\ 0 & 0 & 0 & E_{-,-\mathbf{k}} \end{pmatrix}. \tag{17}
$$

Thus, eigenenergies $E_{+,\mathbf{k}}, E_{-,\mathbf{k}}, -E_{+,\mathbf{k}}$, and $-E_{-,\mathbf{k}}$ are obtained by diagonalizing $\Sigma\mathcal{M}_{\mathbf{k}}$, leading to the dispersion relation given by eq. (3) in the main text.

SUPPLEMENTARY NOTE 4. CALCULATION OF BERRY CONNECTION

In this section, we start by determining $T_{\mathbf{k}}$ and then derive the Berry connection of each subband from the Hamiltonian $\mathcal{M}_{\mathbf{k}}$ (Supplementary eq. (15)). By diagonalizing $\Sigma\mathcal{M}_{\mathbf{k}}$, eigenvectors for each eigenenergy are determined as

$$\begin{aligned}
E = \ & E_{+,\mathbf{k}} \equiv \sqrt{J^2 + 2J|\mathbf{d}|} : \\
\mathbf{t}_{++,\mathbf{k}} = \ & \frac{1}{\Delta_{\mathbf{k}}^{+}} \begin{pmatrix} A_{\mathbf{k}}(|\mathbf{d}| + d_z) \\ A_{\mathbf{k}}(d_x + id_y) \\ B_{\mathbf{k}}(|\mathbf{d}| + d_z) \\ B_{\mathbf{k}}(d_x + id_y) \end{pmatrix}, \\
E = \ & E_{-,\mathbf{k}} \equiv \sqrt{J^2 - 2J|\mathbf{d}|} : \\
\mathbf{t}_{+-,\mathbf{k}} = \ & \frac{1}{\Delta_{\mathbf{k}}^{-}} \begin{pmatrix} C_{\mathbf{k}}(-d_x + id_y) \\ C_{\mathbf{k}}(|\mathbf{d}| + d_z) \\ D_{\mathbf{k}}(-d_x + id_y) \\ D_{\mathbf{k}}(|\mathbf{d}| + d_z) \end{pmatrix}, \\
E = \ & -E_{-,-\mathbf{k}} = -\sqrt{J^2 - 2J|\mathbf{d}|} : \\
\mathbf{t}_{-+,-\mathbf{k}} = \ & \frac{1}{\Delta_{\mathbf{k}}^{-}} \begin{pmatrix} D_{\mathbf{k}}(-d_x + id_y) \\ D_{\mathbf{k}}(|\mathbf{d}| + d_z) \\ C_{\mathbf{k}}(-d_x + id_y) \\ C_{\mathbf{k}}(|\mathbf{d}| + d_z) \end{pmatrix}, \\
E = \ & -E_{+,-\mathbf{k}} = -\sqrt{J^2 + 2J|\mathbf{d}|} : \\
\mathbf{t}_{--,-\mathbf{k}} = \ & \frac{1}{\Delta_{\mathbf{k}}^{+}} \begin{pmatrix} B_{\mathbf{k}}(|\mathbf{d}| + d_z) \\ B_{\mathbf{k}}(d_x + id_y) \\ A_{\mathbf{k}}(|\mathbf{d}| + d_z) \\ A_{\mathbf{k}}(d_x + id_y) \end{pmatrix},
\end{aligned} \tag{18}$$

where

$$\begin{aligned}
A_{\mathbf{k}} = \ & J + \sqrt{J^2 + 2J|\mathbf{d}|}, \\
B_{\mathbf{k}} = \ & J - \sqrt{J^2 + 2J|\mathbf{d}|}, \\
C_{\mathbf{k}} = \ & J + \sqrt{J^2 - 2J|\mathbf{d}|}, \\
D_{\mathbf{k}} = \ & J - \sqrt{J^2 - 2J|\mathbf{d}|}, \\
\Delta_{\mathbf{k}}^{+2} = \ & 2(A_{\mathbf{k}}^2 - B_{\mathbf{k}}^2)|\mathbf{d}|(|\mathbf{d}| + d_z) \\
= \ & 8|\mathbf{d}|(|\mathbf{d}| + d_z)J\sqrt{J^2 + 2J|\mathbf{d}|}, \\
\Delta_{\mathbf{k}}^{-2} = \ & 2(C_{\mathbf{k}}^2 - D_{\mathbf{k}}^2)|\mathbf{d}|(|\mathbf{d}| + d_z) \\
= \ & 8|\mathbf{d}|(|\mathbf{d}| + d_z)J\sqrt{J^2 - 2J|\mathbf{d}|}.
\end{aligned} \tag{19}$$

Thus, a paraunitary matrix can be constructed as $T_{\mathbf{k}} = (\mathbf{t}_{++,\mathbf{k}}, \mathbf{t}_{+-,\mathbf{k}}, \mathbf{t}_{-+,-\mathbf{k}}, \mathbf{t}_{--,-\mathbf{k}})$. Note that this definition is not valid and a different gauge should be selected for $\mathbf{d} = (0, 0, -d)(d > 0)$. The following discussion can be also applied to eigenvectors with a different gauge.

The Berry connection can be defined by the following equation [10, 11],

$$A_{j\mu,\mathbf{k}} = -i\mathrm{Tr}\left[\Gamma_j \Sigma T_{\mathbf{k}}^{\dagger} \Sigma \frac{\partial T_{\mathbf{k}}}{\partial k_{\mu}}\right], \tag{20}$$

where Γ_j is a diagonal matrix, the j-th diagonal component of which is 1 while others are zero, and $\mu = x, y$. From Supplementary eq. (20), the Berry connection of each subband for $\mu = x$ can also be rewritten as

$$\begin{aligned} A_{++,\mathbf{k}} &= -i\mathbf{t}^{\dagger}_{++,\mathbf{k}}\Sigma\frac{\partial \mathbf{t}_{++,\mathbf{k}}}{\partial k_x}, \\ A_{+-,\mathbf{k}} &= -i\mathbf{t}^{\dagger}_{+-,\mathbf{k}}\Sigma\frac{\partial \mathbf{t}_{+-,\mathbf{k}}}{\partial k_x}, \\ A_{-+,\mathbf{k}} &= i\mathbf{t}^{\dagger}_{-+,-\mathbf{k}}\Sigma\frac{\partial \mathbf{t}_{-+,-\mathbf{k}}}{\partial k_x}, \\ A_{--,\mathbf{k}} &= i\mathbf{t}^{\dagger}_{--,-\mathbf{k}}\Sigma\frac{\partial \mathbf{t}_{--,-\mathbf{k}}}{\partial k_x}. \end{aligned} \tag{21}$$

Substituting Supplementary eqs. (18) for Supplementary eqs. (21) leads to

$$\begin{aligned} A_{++,\mathbf{k}} &= \frac{1}{2|\mathbf{d}|(|\mathbf{d}|+d_z)}\left(d_x\frac{\partial d_y}{\partial k_x} - d_y\frac{\partial d_x}{\partial k_x}\right) + \cdots, \\ A_{+-,\mathbf{k}} &= -\frac{1}{2|\mathbf{d}|(|\mathbf{d}|+d_z)}\left(d_x\frac{\partial d_y}{\partial k_x} - d_y\frac{\partial d_x}{\partial k_x}\right) + \cdots, \\ A_{-+,\mathbf{k}} &= A_{+-,\mathbf{k}}, A_{--,\mathbf{k}} = A_{++,\mathbf{k}}. \end{aligned} \tag{22}$$

The first real term corresponds to the phase change of the eigenvector along the Brillouin zone, while the remaining of imaginary terms omitted in Supplementary eq. (22) are due to band deformation. For a one-dimensional system, the total phase change across the Brillouin zone corresponds to the Zak phase [12]:

$$\gamma_j = -\int_{\mathrm{BZ}} A_{j,\mathbf{k}} = -\int_{\mathrm{BZ}} \mathrm{Re}A_{j,\mathbf{k}}. \tag{23}$$

Under $d_z = 0$, $\mathbf{d}$ can be represented by $(|\mathbf{d}|\cos\theta, -|\mathbf{d}|\sin\theta, 0)$, leading to

$$\begin{aligned} \mathrm{Re}(A_{++,\mathbf{k}}) &= -\frac{1}{2}\frac{\partial\theta}{\partial k_x}, \\ \gamma_{++} &= -\int_{\mathrm{BZ}} A_{++,\mathbf{k}} = n\pi, \\ \gamma_{++} &= -\gamma_{+-} = -\gamma_{-+} = \gamma_{--}, \end{aligned} \tag{24}$$

where the integer n represents the winding number. The exactly same form can be derived from $\mathcal{M}'_{\mathbf{k}} = J\mathbf{1} + \mathbf{d}\cdot\boldsymbol{\sigma}$ for an arbitrary gauge, indicating that topological properties are unchanged even if pair creation and annihilation terms are present.

For triplon bands in $Ba_2CuSi_2O_6Cl_2$, the Berry connection can be obtained from $\mathbf{d} = (\mathrm{Re}\Lambda_{\mathbf{k}}, -\mathrm{Im}\Lambda_{\mathbf{k}}, 0)$ as

$$\begin{aligned} A_{++,\mathbf{k}} &= -\frac{i}{2}\frac{\Lambda_{\mathbf{k}}}{|\Lambda_{\mathbf{k}}|}\frac{\partial}{\partial k_x}\left(\frac{\Lambda^*_{\mathbf{k}}}{|\Lambda_{\mathbf{k}}|}\right) \\ &\quad + i\left\{\frac{2B_{\mathbf{k}}}{\Delta^+_{\mathbf{k}}}\frac{\partial}{\partial k_x}\left(\frac{B_{\mathbf{k}}}{\Delta^+_{\mathbf{k}}}\right) - \frac{2A_{\mathbf{k}}}{\Delta^+_{\mathbf{k}}}\frac{\partial}{\partial k_x}\left(\frac{A_{\mathbf{k}}}{\Delta^+_{\mathbf{k}}}\right)\right\}, \\ A_{+-,\mathbf{k}} &= -\frac{i}{2}\frac{\Lambda^*_{\mathbf{k}}}{|\Lambda_{\mathbf{k}}|}\frac{\partial}{\partial k_x}\left(\frac{\Lambda_{\mathbf{k}}}{|\Lambda_{\mathbf{k}}|}\right) \\ &\quad + i\left\{\frac{2D_{\mathbf{k}}}{\Delta^-_{\mathbf{k}}}\frac{\partial}{\partial k_x}\left(\frac{D_{\mathbf{k}}}{\Delta^-_{\mathbf{k}}}\right) - \frac{2C_{\mathbf{k}}}{\Delta^-_{\mathbf{k}}}\frac{\partial}{\partial k_x}\left(\frac{C_{\mathbf{k}}}{\Delta^-_{\mathbf{k}}}\right)\right\}, \\ A_{--,\mathbf{k}} &= A_{++,\mathbf{k}}, \\ A_{-+,\mathbf{k}} &= A_{+-,\mathbf{k}}, \end{aligned} \tag{25}$$

which leads to the Zak phase quantized into $\gamma_{++} = -\gamma_{+-} = -\gamma_{-+} = \gamma_{--} = \pm\pi$ irrespective of k_y.

SUPPLEMENTARY NOTE 5. CALCULATION OF AN ENERGY SPECTRUM

As discussed in the main text, edge states should appear at the end of the a-direction from an analogy with a coupled SSH model [13]. To confirm this, an energy spectrum of the present model is calculated by imposing open boundary conditions along the a-direction. For simplicity, Fourier-transformed operators are defined under periodic boundary conditions along the b-direction as

$$\begin{aligned} t^{\dagger}_{\alpha mn} &= \sqrt{\frac{2}{N_b}} \sum_{k_y} e^{ik_y y_{mn}} t^{\dagger}_{\alpha m k_y}, \\ t_{\alpha mn} &= \sqrt{\frac{2}{N_b}} \sum_{k_y} e^{-ik_y y_{mn}} t_{\alpha m k_y}, \end{aligned} \tag{26}$$

where N_b is the number of chains along b. Substituting Supplementary eqs. (26) for Supplementary eqs. (9) leads to

$$\mathcal{H} = \frac{1}{2} \sum_{k_y} \sum_{\alpha=x,y,z} \mathbf{m}^{\dagger}_{\alpha k_y} \begin{pmatrix} J\mathbf{1} + \mathbf{X}_{k_y} & \mathbf{X}_{k_y} \\ \mathbf{X}_{k_y} & J\mathbf{1} + \mathbf{X}_{k_y} \end{pmatrix} \mathbf{m}_{\alpha k_y}, \tag{27}$$

where $\mathbf{m}_{\alpha k_y}$ represents a $4N_a$ $(2N_a \equiv N/N_b)$ component vector

$$\begin{aligned} \mathbf{m}_{\alpha k_y} \equiv (& t^{1,\dagger}_{\alpha m k_y}, t^{1,\dagger}_{\alpha m k_y}, \cdots, t^{N_a,\dagger}_{\alpha m k_y}, t^{N_a,\dagger}_{\alpha m k_y}, \\ & t^{1}_{\alpha m -k_y}, t^{1}_{\alpha m -k_y}, \cdots, t^{N_a}_{\alpha m -k_y}, t^{N_a}_{\alpha m -k_y})^T, \end{aligned} \tag{28}$$

and $\mathbf{1}$ is an $N_a \times N_a$ identity matrix. $\mathbf{X}_{k_y}$ is a $2N_a \times 2N_a$ matrix defined as

$$\mathbf{X}_{k_y} = \begin{pmatrix} 0 & J_{k_y} & 0 & J^{\mathrm{A}'} & 0 & 0 & \cdots & 0 & 0 \\ J_{k_y} & 0 & J^{\mathrm{A}} & 0 & 0 & 0 & \cdots & 0 & 0 \\ 0 & J^{\mathrm{A}} & 0 & J_{k_y} & 0 & J^{\mathrm{A}'} & \cdots & 0 & 0 \\ J^{\mathrm{A}\prime} & 0 & J_{k_y} & 0 & J^{\mathrm{A}} & 0 & \cdots & 0 & 0 \\ 0 & 0 & 0 & J^{\mathrm{A}} & 0 & J_{k_y} & \cdots & 0 & 0 \\ 0 & 0 & J^{\mathrm{A}'} & 0 & J_{k_y} & 0 & \cdots & 0 & 0 \\ \vdots & \vdots & \vdots & \vdots & \vdots & \vdots & \ddots & \vdots & \vdots \\ 0 & 0 & 0 & 0 & 0 & 0 & \cdots & 0 & J_{k_y} \\ 0 & 0 & 0 & 0 & 0 & 0 & \cdots & J_{k_y} & 0 \end{pmatrix}, \tag{29}$$

where $J_{k_y} = 2J_B \cos(k_y b/2)$. The energy spectrum shown in Figure 5 is obtained by diagonalizing the matrix with $N_a = 100$ for each k_y. Note that edge states exhibit a very weak dispersion, as shown in Supplementary Figure 3, which becomes even weaker with increasing N_a. While pair creation and annihilation terms make the bulk energy spectrum asymmetric above and below energy J (Supplementary Figure 3a), they do not affect the dispersion of the edge modes (Supplementary Figure 3b).

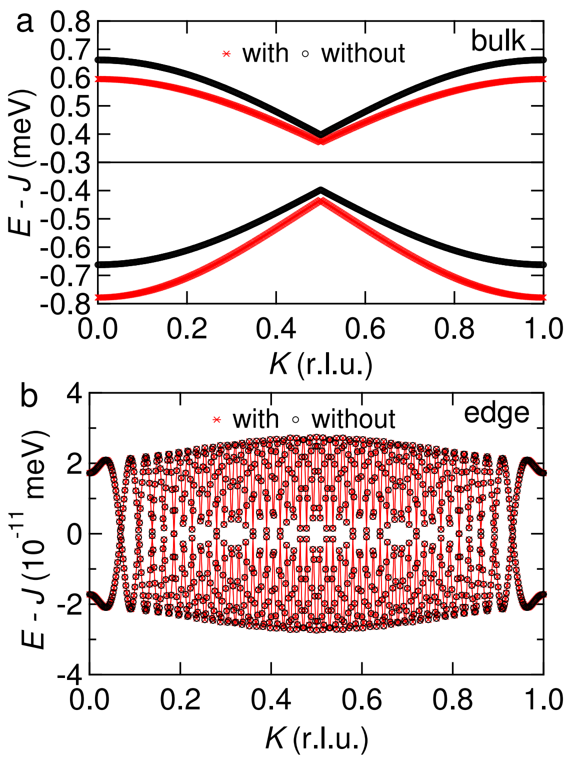

Supplementary Figure 3. **a,b.** The dispersion of (**a**) the highest- and lowest energy modes of bulk bands and (**b**) two edge modes along the *b*-axis. The energy represents the difference with respect to the intradimer exchange J. Red crosses and black circles indicate the modes derived from Hamiltonian with ($\mathcal{M}_{\mathbf{k}}$) and without ($\mathcal{M}'_{\mathbf{k}}$) pair creation and annihilation terms, respectively.

SUPPLEMENTARY REFERENCES

[1] Tanaka, H. *et al.* Almost perfect frustration in the dimer magnet $Ba_2CoSi_2O_6Cl_2$. *J. Phys. Soc. Jpn.* **83**, 103701 (2014).

[2] Sparta, K. M. & Roth, G. Reinvestigation of the structure of $BaCuSi_2O_6$ – evidence for a phase transition at high temperature. *Acta Crystallogr. B* **60**, 491 (2004).

[3] Sasago, Y., Uchinokura, K., Zheludev, A. & Shirane, G. Temperature-dependent spin gap and singlet ground state in $BaCuSi_2O_6$. *Phys. Rev. B* **55**, 8357 (1997).

[4] Okada, M. *et al.* Quasi-two-dimensional Bose-Einstein condensation of spin triplets in the dimerized quantum magnet $Ba_2CuSi_2O_6Cl_2$. *Phys. Rev. B* **94**, 094421 (2016).

[5] Sebastian, S. E. *et al.* Dimensional reduction at a quantum critical point. *Nature* **441**, 617 (2006).

[6] Samulon, E. C. *et al.* Low-temperature structural phase transition and incommensurate lattice modulation in the spin-gap compound $BaCuSi_2O_6$. *Phys. Rev. B* **73**, 100407 (2006).

[7] Sachdev, S. & Bhatt, R. N. Bond-operator representation of quantum spins: Mean-field theory of frustrated quantum Heisenberg antiferromagnets. *Phys. Rev. B* **41**, 9323 (1990).

[8] Matsumoto, M., Normand, B., Rice, T. M. & Sigrist, M. Magnon dispersion in the field-induced magnetically ordered phase of $TlCuCl_3$. *Phys. Rev. Lett.* **869**, 077203 (2002).

[9] Matsumoto, M., Normand, B., Rice, T. M. & Sigrist, M. Field- and pressure-induced magnetic quantum phase transitions in $TlCuCl_3$. *Phys. Rev. B* **69**, 054423 (2004).

[10] Shindou, R., Matsumoto, R., Murakami, S. & Ohe, J. Topological chiral magnonic edge mode in a magnonic crystal. *Phys. Rev. B* **87**, 144427 (2013).

[11] Murakami, S. & Okamoto, A. Thermal Hall effect of magnons. *J. Phys. Soc. Jpn.* **86**, 011010 (2017).

[12] Zak, J. Berry's phase for energy bands in solids. *Phys. Rev. Lett.* **62**, 23 (1989).

[13] Li, C., Lin, S., Zhang, G. & Song, Z. Topological nodal points in two coupled Su-Schrieffer-Heeger chains. *Phys. Rev. B* **96**, 125418 (2017).
